# Utilization of maternal healthcare among adolescent mothers in urban India: evidence from DLHS-3

**DOI:** 10.7717/peerj.592

**Published:** 2014-11-04

**Authors:** Aditya Singh, Abhishek Kumar, Pragya Pranjali

**Affiliations:** 1 Global Health and Social Care Unit, School of Health Sciences and Social Work, University of Portsmouth, Portsmouth, United Kingdom; 2 International Institute for Population Sciences, Mumbai, India; 3 Social Research Institute - IRMB International, New Delhi, India

**Keywords:** DLHS-3, Urban India, Adolescent health, Maternal health, Antenatal care, Safe delivery, Postnatal care

## Abstract

**Background.** Low use of maternal healthcare services is one of the reasons why maternal mortality is still considerably high among adolescents mothers in India. To increase the utilization of these services, it is necessary to identify factors that affect service utilization. To our knowledge, no national level study in India has yet examined the issue in the context urban adolescent mothers. The present study is an attempt to fill this gap.

**Data and Methods.** Using information from the third wave of District Level Household Survey (2007–08), we have examined factors associated with the utilization of maternal healthcare services among urban Indian married adolescent women (aged 13–19 years) who have given live/still births during last three years preceding the survey. The three outcome variables included in the analyses are ‘full antenatal care (ANC)’, ‘safe delivery’ and ‘postnatal care within 42 days of delivery’. We have used Chi-square test to determine the difference in proportion and the binary logistic regression to understand the net effect of predictor variables on the utilization of maternity care.

**Results.** About 22.9% of mothers have received full ANC, 65.1% of mothers have had at least one postnatal check-up within 42 days of pregnancy. The proportion of mother having a safe delivery, i.e., assisted by skilled personnel, is about 70.5%. Findings indicate that there is considerable amount of variation in use of maternity care by educational attainment, household wealth, religion, parity and region of residence. Receiving full antenatal care is significantly associated with mother’s education, religion, caste, household wealth, parity, exposure to healthcare messages and region of residence. Mother’s education, full antenatal care, parity, household wealth, religion and region of residence are also statistically significant in case of safe delivery. The use of postnatal care is associated with household wealth, woman’s education, full antenatal care, safe delivery care and region of residence.

**Conclusion.** Several socioeconomic and demographic factors affect the utilization of maternal healthcare services among urban adolescent women in India. Promoting the use of family planning, female education and higher age at marriage, targeting vulnerable groups such as poor, illiterate, high parity women, involving media and grass root level workers and collaboration between community leaders and health care system could be some important policy level interventions to address the unmet need of maternity services among urban adolescents.

## Introduction

According to an estimate, around 16 million adolescent women (aged 15–19) give birth every year around the world and most of these births (about 95%) are concentrated in middle and low income countries (World Health Organization, 2012). Childbirth in adolescence is often risky. It is associated with a host of life threatening adverse health outcomes such as high risk of premature delivery, delivery and postnatal complications, unsafe abortion complications, and obstetric fistula etc. (Christiansen, Gibbs & Chandra-Mouli, 2013; Omar et al., 2010; Singh, Kumar & Kumar, 2013; Wang, Wang & Lee, 2012; Wilson et al., 2012; World Health Organization, 2007). Hence, it is not surprising that despite accounting for only 11% births worldwide, adolescent women carry 23% of overall burden of disease (in terms of disability adjusted life years) due to pregnancy and childbirth among women of all ages (Gore et al., 2011; Mangiaterra et al., 2008). Complications of pregnancy and childbirth are also among the leading causes of death among women aged 15–19 years (World Health Organization, 2014). India is no exception in this regard. Despite a substantial improvement in maternal mortality in last two decades, the proportion of adolescent maternal deaths to total maternal deaths is still around 10%.

Most maternal deaths are preventable if mothers receive essential healthcare before, during, and after childbirth (Save the Children, 2013). India, being a signatory to many regional and international agreements including the Alma-Ata Declaration (1978), International Conference on Population and Development, Cairo (1994) and the Millennium Declaration (2000), bears a legal obligation to make sure that women do not die or suffer complications from preventable pregnancy-related causes (United Nations, 1994; United Nations, 2000). Over past decades, the Government of India has implemented several policies and programs such as Child Survival and Safe Motherhood Programme, 1992; Reproductive and Child Health (RCH) Program, 1997; National Population Policy, 2000; and National Urban Health Mission, 2013–2017 to reduce the burden of maternal mortality and improve maternal health. As a result, the overall utilization of maternal health care services in India has improved over time, however, the level of uptake is still considered low among adolescent mothers (Kumar et al., 2013). It is not only true for rural adolescent mothers but also for urban adolescent mothers (Table 1).

**Table 1:** Maternal healthcare utilization among married mothers of different age groups in urban India, 2007–08.

Healthcare utilization indicators	13–19	20–24	25–29	30–49	15–49
Four or more antenatal care visits	46.9	54.8	56.1	49.0	53.5
IFA adequate for 100 days	40.1	43.2	46.6	48.5	45.0
Two or more TT injections	77.5	81.3	80.7	73.4	79.3
Full antenatal care^a^	22.9	29.7	31.6	28.2	29.5
Safe delivery^b^	70.5	77.5	78.4	72.4	76.3
Home delivery	36.3	28.0	26.5	33.1	29.1
Postnatal check-up within 48 h	61.5	68.6	70.3	65.1	68.0
Postnatal check-up within 2 weeks	61.6	69.1	70.9	65.7	68.5
Postnatal check-up within 42 days	65.1	71.3	72.8	67.5	70.6

**Notes.**

a A mother is considered to have received full antenatal care only when she had a minimum of three antenatal check-ups, two tetanus toxoid injections, and iron and folic acid tablets for 90 days or more during her pregnancy period.

b A delivery conducted either in a medical institution or home deliveries assisted by doctor/nurse/Lady Health Visitor (LHV)/Auxiliary Nurse Midwife (ANM)/other qualified health professionals.

The utilisation of maternal healthcare is a complex phenomenon influenced by several factors. Several studies from developing countries have recognised socioeconomic factors and service delivery environment as important determinants of healthcare utilization. Quality of care, distance to health facility, lack of transport, women’s low social status, age, caste, religion, educational level, economic status of the household, lack of autonomy and decision-making power and cultural norms are some of the factors that have been found to be associated with the utilization maternal care services use in different settings (Edmonds, Paul & Sibley, 2012; Gabrysch & Campbell, 2009; Joshi et al., 2014; Masters et al., 2013; Nair et al., 2014; Sepehri et al., 2008; Sharma et al., 2014; Tsegay et al., 2013; Yamashita et al., 2014). Similarly, several studies in India have also examined factors affecting maternal care utilisation (Arokiasamy & Pradhan, 2013; Jat, Ng & San Sebastian, 2011; Mistry, Galal & Lu, 2009; Singh, Rai & Singh, 2012; Singh et al., 2012; Sunil, Rajaram & Zottarelli, 2006); however, none of these studies have focused on urban adolescent mothers.

In India, the urban population growth has outpaced the growth of essential public services such as sanitation, healthcare, housing etc. Together with rising poverty, it has forced a considerable chunk of urban population to live in difficult physical and social environment which has adverse health impacts (Gupta, Arnold & Lhungdim, 2009; Ministry of Urban Development, 2008; Montgomery, 2009). Urban adolescent girls too have to face many social and health related challenges such as early marriage, unwanted and early age pregnancy, and illegal and unsafe abortion (Jejeebhoy et al., 2009; Mehra & Agrawal, 2004; Moore et al., 2009). The risk of early pregnancy is further exacerbated by poverty, disruption in schooling and education, and inadequate access to family planning services (Banerjee et al., 2009). Early childbearing has adverse micro and macro level impacts—i.e., it not only affects the health of the mother and her child but it also has repercussions for the society as a whole (Mangiaterra et al., 2008). In order to ensure better health for adolescent urban mothers, it is necessary to examine their healthcare needs and identify the barriers in access to healthcare services. It can help the government in designing appropriate, context-relevant program and policy responses under recently launched National Urban Health Mission (NUHM) which is currently in initial phases of implementation across 779 cities and towns with population more than 50,000 (Government of India, 2013). Using the most recent data from the third wave of District Level Household Survey (2007–08), the present study, therefore, aims to examine the factors associated with selected indicators of utilization of maternal healthcare services with reference to adolescent mothers (13–19 years) living in urban India.

## Data and Methods

### Data

We use data from the third round of the District Level Household Survey (DLHS-3) conducted during 2007–08. The DLHS is a nationally representative and one of largest ever demographic surveys conducted in India. It covers all states and union territories of India except Nagaland. The basic aim of DLHS-3 is to provide reliable estimates of maternal and child health, family planning and other reproductive health indicators at district level (International Institute for Population Sciences, 2010).

### Sampling design and study size

**Figure 1:**
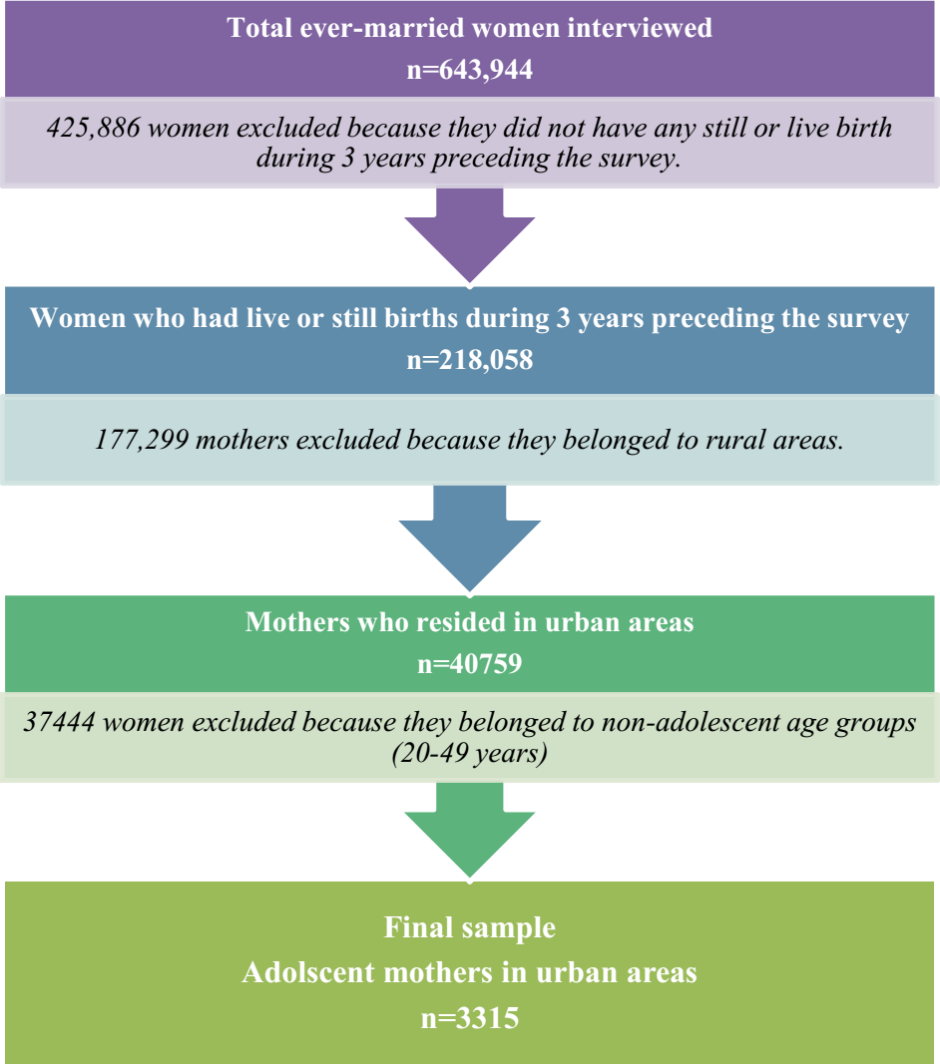
Flow chart showing the process of selection of urban adolescent mothers’ sample.

DLHS-3 adopted a multi-stage stratified systematic sampling design. Detailed information about sampling employed in this survey can be obtained from the national report of DLHS-3. The survey interviews 643,944 ever-married women aged 13–29 years from 720,320 sampled household (about 78% from rural and 22% from urban areas) spanning 601 districts of India. The overall response rate for ever-married women at the national level is 89%. Out of these 643,944 ever-married women, a total of 201,058 have had a still or live birth during three years preceding the survey (International Institute for Population Sciences, 2010). Among these, only 40,759 women live in urban areas. After excluding 37,444 women who belong to non-adolescent age groups (20–49), the sample is reduced to 3,315 adolescent women. These 3,315 adolescent women form the basis of our analysis in this paper (Fig. 1).

### Dependent variables

We have used ‘full antenatal care’, ‘safe delivery’ and ‘postnatal care’ as indicators of maternal healthcare utilization. They have been defined on the basis of the guidelines provided by the World Health Organization. A woman is considered to have received full antenatal care only when she has had at least three antenatal check-ups, two tetanus toxoid injections, and iron and folic acid tablets/syrup for 90 days or more during her pregnancy (World Health Organization, 2006). A delivery conducted either in a medical institution or home deliveries assisted by a doctor/nurse/Lady Health Visitor (LHV)/Auxiliary Nurse Midwife (ANM)/other qualified health professionals is considered a safe delivery in this study. A woman is considered to have received postnatal care if she had a postnatal check-up within 42 days after delivery (World Health Organization, 1998).

### Independent variables

We have considered a range of socio-economic and demographic predictors such as woman’s education, husband’s education, religion, caste, exposure to mass media, economic status, employment, parity, and region of residence. The choice of these variables is basically guided by existing literature on maternity care utilization (Babalola & Fatusi, 2009; Gabrysch & Campbell, 2009; Gage & Guirlène Calixte, 2006; Ghosh, 2006; Haque, 2014; Jat, Ng & San Sebastian, 2011; Joshi et al., 2014; Kesterton et al., 2010; Navaneetham & Dharmalingam, 2002; Rahman, Haque & Zahan, 2011; Reynolds, Wong & Tucker, 2006; Saroha, Altarac & Sibley, 2014; Say & Raine, 2007; Singh, Rai & Singh, 2012; Singh et al., 2012; Singh et al., 2014; Sunil, Rajaram & Zottarelli, 2006).

The education level of the woman (mother) and her husband is defined using the number of years of schooling. The variable has five categories: illiterate, literate but below primary, primary but below middle school, middle but below high school, and high school and above. The type of work in which the mother was engaged in the last year from the date of interview is considered her employment. The variable has three categories—unemployed, professional/service/production worker, and agricultural worker/farmer/labourers.

The entire sample of mothers can be divided in four social groups namely ‘Others’ (General), Scheduled Castes (SCs), Scheduled Tribes (STs) and Other Backward Classes (OBCs). These are the official categories used by the Government of India. The social system in India is characterised by numerous castes irrespective of religion. The castes deemed elite by the Indian society in the past are now officially classified as “General” or “Others”. Apart from them, other socioeconomically deprived communities have been categorized as ‘lower castes’. These communities (lower castes), based on their status in society, are further divided in to two categories—Other Backward Classes and Scheduled Castes. Scheduled Castes comprise mainly those groups that are considered to be ‘untouchables’. The category of ‘Other Backward Classes’ comprises castes other than elites and untouchables. Scheduled Tribes are the groups that do not practice the caste system and live in hilly, forested and remote areas largely secluded from mainstream society of India. STs and SCs make up around 7% and 16% of the total population of India, respectively. The estimates of OBC population vary according to the source. For this purpose, the Government of India follows the report submitted in 1980 by the Second Backward Classes Commission, which puts the figure as high as 52% of the total population. The rest of them are officially categorised as ‘General’/‘Others’ and make up around 25% of the total population (Ramaiah, 1992). There are three categories for religion—Hindu, Muslim, and Other (includes Sikhism, Christianity, Buddhism, Jainism, and other religions).

Wealth index is generally used as a proxy for the economic status of the household (Filmer & Pritchett, 2001; Montgomery et al., 2000). In this study, it is a composite index of household amenities and assets with five categories—poorest, poorer, middle, richer, and richest. The variable for exposure to maternity care related messages has been constructed for both antenatal care and safe delivery care separately. It has four categories—not heard/seen/read any messages; heard/seen/read from mass media (TV/radio/newspaper/books/magazine/hoardings/pamphlets/posters); received message through interpersonal communication (drama/song/dance performance/street play/puppet show/exhibition/mela/group meeting/doctor/ANM/ASHA/friends/relatives); and both (mass media and interpersonal communication). It should be noted that no question has been asked in the survey about the exposure to postnatal care messages.

Regional variation in the use of maternity care has been documented by many previous studies (Navaneetham & Dharmalingam, 2002; Singh, Rai & Singh, 2012). To adjust for the regional variation in the utilization of maternal healthcare, we have included ‘region of residence’ as an explanatory variable in the analysis. For this purpose, we have divided India into six regions based on geographic and cultural settings. The six regions consist of North (Jammu and Kashmir, Himachal Pradesh, Punjab, Haryana, Rajasthan, Delhi and Uttaranchal), Central (Uttar Pradesh, Madhya Pradesh and Chhattisgarh), East (Bihar, Jharkhand, West Bengal and Orissa), North-East (Arunachal Pradesh, Assam, Manipur, Meghalaya, Mizoram, Nagaland, Sikkim and Tripura), West (Gujarat, Maharashtra and Goa), and South (Andhra Pradesh, Karnataka, Kerala and Tamil Nadu).

### Statistical analysis

We used both bivariate and multivariate analyses to identify factors associated with maternal healthcare utilization among adolescent mothers in urban India. Chi-square test is used to determine the difference in proportions of the service utilization across selected socioeconomic and demographic characteristics. Binary logistic regression is applied to understand the net effect of predictor variables on the utilization of maternal health care services—full antenatal care, safe delivery and postnatal care. We have chosen logistic regression because the response variables in our study are of dichotomous (i.e., binary) nature. Only those predictor variables that are found significant in chi-square test are included in the final binary logistic regression model. The results of logistic regression are presented in the form of estimated odds-ratios with *p*-values and 95% confidence intervals (CI).

## Results

### Profile of the respondents

Table 2 presents the weighted percentage distribution of adolescent women who have delivered their last child during last three years preceding the survey by selected background characteristics. The majority of adolescent mothers (81.8%) had given birth after 17 years of age. About 28% of adolescent mothers were illiterate and the majority of them were Hindu. Among social groups, 46.7% of mothers belonged to OBC. About 83% of mothers were unemployed and 17.4% were married to an illiterate husband. About 7.3% of mothers reported no exposure to antenatal care messages while about 15.6% had no exposure to safe delivery care messages. The proportion of mothers belonging to the poorest and poorer wealth quintiles was about 4.6% and 9.2%, respectively.

**Table 2:** Percentage distribution of adolescent mothers by background characteristics, DLHS-3 (2007–08), urban India.

Background characteristics	%	*N* ^a^
**Wealth quintiles**		
Lowest	4.62	153
Second	9.20	305
Middle	17.07	566
Fourth	36.14	1,198
Highest	32.97	1,093
**Husband’s education**		
Illiterate	17.35	575
Below 5 years	7.03	233
6–8 years	20.30	673
9–10 years	21.24	704
11 and above	34.09	1,130
**Mother’s education**		
Illiterate	27.87	924
Below 5 years	7.00	232
6–8 years	22.96	761
9–10 years	19.82	657
11 and above	22.35	741
**Caste/Tribe**		
Scheduled tribes	8.04	680
Scheduled castes	20.96	261
Other backward castes	46.72	1,516
General	24.28	788
**Religion**		
Hindu	70.05	2,322
Muslim	23.5	779
Others	6.46	214
**Mother’s employment**		
Unemployed	82.66	2,736
Professional/service/production worker	7.79	258
Agricultural worker/labourer/farmer	9.55	316
**Age of the mother**		
13–17	18.16	602
18–19	81.84	2,713
**Parity**		
1	78.83	2581
2	17.75	581
3 +	3.42	112
**Exposure to antenatal care messages**		
No exposure	7.33	243
Only mass media	9.47	314
Only interpersonal communication	41.09	1,362
Both of the above	42.11	1,396
**Exposure to safe delivery messages**		
No exposure	15.66	519
Only mass media	9.08	301
Only interpersonal communication	38.76	1,285
Both of the above	36.5	1,210
**Region**		
North	10.95	363
Central	36.02	1,194
North East	8.57	284
East	10.77	357
West	13.54	449
South	20.15	668
**Full antenatal care**		
No	77.02	2,551
Yes	22.98	761
**Safe delivery**		
No	29.51	978
Yes	70.49	2,336

**Notes.**

Note: All ‘n’ are unweighted.

a The total may not be equal due to some missing cases.

### Differentials in the utilization of maternal healthcare services

We examined bivariate differentials to explore how utilization of maternity services varies across selected socioeconomic and demographic characteristics. Table 3 presents weighted percentage of women who utilized maternity services by background characteristics. Overall, 22.9% of mothers received full antenatal care, 70.5% utilized safe delivery care and 65.1% had a postnatal check-up.

**Table 3:** Usage pattern of maternal healthcare among adolescent mothers by background characteristics, DLHS-3 (2007–08), urban India.

Background characteristics	Antenatal care	Safe delivery	Postnatal care
**Wealth quintiles**	(84.8)^***^	(202.4)^***^	(176.3)^***^
Lowest	4.58	41.83	37.33
Second	13.77	50.82	47.70
Middle	16.96	62.83	58.11
Fourth	24.37	72.45	65.04
Highest	29.72	81.79	77.47
**Religion**	(7.2)^**^	(18.2)^***^	(3.1)ns
Hindu	23.28	71.22	64.15
Muslim	20.44	65.73	66.93
Others	28.97	79.91	68.60
**Caste/tribe**	(11.3)^**^	(6.3)^*^	(17.3)^***^
Scheduled tribes	18.41	68.19	62.28
Scheduled castes	25.29	70.50	59.29
Other backward castes	24.67	70.25	64.60
General	22.65	73.98	70.75
**Husband’s education**	(73.2)^***^	(159.6)^***^	(93.6)^***^
Illiterate	12.70	52.44	50.79
Below primary	21.03	63.95	57.83
Primary but below middle	21.99	70.13	64.71
Middle but below high school	20.88	70.17	65.48
High school and above	30.52	81.42	73.77
**Mother’s education**	(201.9)^***^	(341.1)^***^	(218.7)^***^
Illiterate	10.28	48.86	46.61
Below primary	16.81	65.95	61.40
Primary but below middle	21.45	74.51	69.35
Middle but below high school	26.83	78.39	71.85
High school and above	38.92	87.72	78.80
**Mother’s employment**	(7.5)^***^	(21.7)^***^	(10.2)^***^
Unemployed	23.23	71.70	65.68
Professional/service/production worker	27.13	72.09	68.75
Agricultural worker/labourer	17.72	59.18	57.32
**Parity**	(20.4)^***^	(95.4)^***^	(36.6)^***^
1	24.90	74.73	68.01
2 +	16.74	55.7	55.62
**Exposure to antenatal care messages**	(50.0)^***^		
No exposure	7.82		
Only through mass media	26.84		
Only through interpersonal communication	20.78		
Both	26.90		
**Exposure to safe delivery care messages**		(133.8)^***^	
No exposure		51.35	
Only through mass media		74.42	
Only through interpersonal communication		69.49	
Both		78.76	
**Full antenatal care**		(198.3)^***^	(197.7)^***^
No		64.37	58.69
Yes		90.92	86.49
**Safe Delivery**			(962.1)^***^
No			25.13
Yes			81.74
**Region**	(382.9)^***^	(260.2)^***^	(321.7)^***^
North	17.96	62.53	63.03
Central	10.98	56.7	50.46
North East	20.07	73.94	56.36
East	14.61	72.55	60.39
West	28.51	83.04	85.07
South	49.10	88.47	85.24
**Urban India**	22.98	70.49	65.08

**Notes.**

Note: Figure in parentheses is the Chi-square statistics; x2 test applied for each variable. Level of significance:

* *p* < 0.10

** *p* < 0.05

*** *p* < 0.01.

The coverage of full antenatal care is low among illiterate mothers (10.3%). Only about 48.9% of mothers with no formal education reported a safe delivery while about 87.7% of those with high school education have chosen to do the same. The proportion of illiterate mothers who received postnatal care within 42 days after the delivery was about 46.6%. In contrast, the same proportion among educated mothers (with ten or more years of schooling) was 78.8%. The utilization of services among adolescent women registers an increase with an increase in their husbands’ years of schooling. The coverage of full antenatal care, safe delivery and postnatal care among women with educated husbands (ten or more years of schooling) was 30.5%, 83.4% and 73.8%, respectively.

Only two-third of Muslim mothers reported using safe delivery services compared to 71% among Hindus. Similarly, postnatal care is utilized more by mothers from other religious groups (69%), followed by Muslims (67%) and Hindus (64%). While the utilization of full antenatal care (19%), safe delivery (68%) is lowest among STs, the postnatal care utilization was lowest among SCs (60%). It should be noted that caste differentials in urban areas were not quite large.

We observed an increase in the level of utilization with increase in household wealth. For instance, only 4.6% of mothers belonging to the poorest wealth quintile reported having full antenatal care, while the same proportion was 29.7% among mothers belonging to the richest wealth quintile. A similar pattern was observed for safe deliveries and postnatal care.

Only about 17.7% and 59.2% of mothers working as agricultural labourer/workers receive full antenatal and safe delivery care, respectively compared to 27.1% and 72.1% of mothers working as professional/service/production workers. About 26.9% of mothers who heard antenatal care messages through both mass media and interpersonal communication received full antenatal care. On the other hand, the corresponding figure for mothers with no mass media exposure was just 7.8%. Similarly, about 74% and 68% of mothers with mass media exposure reported using delivery and postnatal care. Fewer adolescent mothers of parity two and above were able to receive full antenatal care (16.7%) and safe delivery (55.7%) compared to mothers of parity one.

The utilization of maternal health care services among mothers from the Southern region is higher than among mothers from other regions. While the highest utilization of full antenatal care (49.1%) is observed in the South, the lowest is among the mothers from the Central region (10.9%). The Central region also fares badly in terms of safe delivery (56.7%) and postnatal care (50.5%).

### Determinants of full antenatal care utilization

Table 4 presents the results of binary logistic regression showing the determinants of utilization of full antenatal care among adolescent mother. Woman’s education, husband’s education, religion, caste, economic status, parity, exposure to antenatal care massages and region of residence turned out to be significant determinants of the utilization of antenatal care services among urban adolescent mothers.

**Table 4:** Odds ratios and 95% confidence intervals (CI) for receiving full antenatal care, DLHS- 3 (2007–08), urban India.

Background characteristics	Odds ratio	*P*-value	95% CI	
			Lower	Upper
**Wealth quintiles**				
Lowest	1.000			
Second	2.524	0.035	1.068	5.963
Middle	2.550	0.026	1.118	5.816
Fourth	3.646	0.002	1.621	8.201
Highest	3.850	0.001	1.688	8.782
**Religion**				
Hindu	1.000			
Muslim	0.731	0.011	0.574	0.931
Others	1.335	0.171	0.882	2.021
**Caste/tribe**				
Scheduled tribes	1.000			
Scheduled castes	1.541	0.046	1.008	2.356
Other backward castes	1.172	0.241	0.899	1.528
General	1.211	0.202	0.902	1.626
**Husband’s education**				
Illiterate	1.000			
Below primary	1.450	0.108	0.921	2.282
Primary but below middle	1.349	0.093	0.951	1.912
Middle but below high school	1.236	0.250	0.861	1.774
High school and above	1.373	0.075	0.969	1.945
**Mother’s education**				
Illiterate	1.000			
Below primary	1.389	0.144	0.894	2.160
Primary but below middle	1.535	0.007	1.127	2.090
Middle but below high school	1.935	0.000	1.402	2.670
High school and above	2.346	0.000	1.680	3.276
**Mother’s employment**				
Unemployed	1.000			
Professional/service/production worker	1.192	0.295	0.858	1.657
Agricultural worker/labourer	1.055	0.766	0.743	1.496
**Parity**				
1	1.000			
2 +	0.710	0.007	0.554	0.910
**Exposure to antenatal care messages**				
No exposure	1.000			
Only through mass media	2.929	0.000	1.643	5.223
Only through interpersonal communication	2.283	0.002	1.355	3.845
Both	2.751	0.000	1.635	4.629
**Region**				
North	1.000			
Central	0.650	0.014	0.461	0.917
North East	0.796	0.363	0.488	1.301
East	0.984	0.942	0.636	1.521
West	1.953	0.000	1.365	2.795
South	4.757	0.000	3.372	6.711

Mothers with middle school education are about two times (OR = 1.935, CI = 1.402–2.670), and mothers with higher education, are about two and half times (OR = 2.346 CI = 1.680–3.276) more likely to receive full antenatal care than illiterate mothers. This is similar to mothers with primary level education (OR = 1.535, CI = 1.127–2.090). Adolescent women with ‘high school or above’ educated husbands are more likely to utilize full antenatal care than women with illiterate husbands (OR = 1.373, CI = 0.969–1.945).

The wealth status exerts a positive effect on the utilization of full antenatal care among urban adolescent mothers. Mothers from richer and richest wealth quintiles are nearly four times (OR = 3.646, CI = 1.621–8.201; OR = 3.850, CI = 1.688–8.782) more likely to receive full antenatal care than mothers belonging to the poorest wealth quintile. Mothers with parity two or more are less likely to receive full antenatal care than mothers with parity one (OR = 0.710, CI = 0.554–0.910).

Region of residence exerts significant influence on use of full ANC services. Adolescent mothers from the West and the South are more likely to receive full antenatal care than mothers from the North. Adolescent mothers in the South and the West are two (OR = 1.953, CI = 1.365–2.795) and four (OR = 4.757, CI = 3.372–6.711) times more likely to use of full antenatal care than their counterparts in the North. The odds of receiving full antenatal care, however, lower among mothers residing in Central region than among those residing in the North.

Adolescent urban mothers who have heard antenatal care messages from mass media or have had interpersonal communication with a health worker or have been lucky enough to have exposure to both are about two times more likely to receive full antenatal care than those who have had no exposure to mass media. Muslim mothers are less likely (OR = 0.731, CI = 0.574–0.931) to receive full antenatal care than their Hindu counterparts. On the other hand, the odds of receiving full antenatal care are higher among SCs (OR = 1.008–2.356) than they are among STs.

### Determinants of safe delivery care

Results of logistic regression analysis for safe delivery care are presented in Table 5. Findings show that wealth index, religion, woman’s education, husband’s education; full antenatal care, birth order and region of residence are statistically significant determinants of safe delivery care utilization.

**Table 5:** Odds ratios and 95% confidence intervals (CI) for safe delivery, DLHS-3 (2007–08), urban India.

Background characteristics	Odds ratio	*P*-value	95% CI	
			Lower	Upper
**Wealth quintiles**				
Lowest	1.000			
Second	1.117	0.627	0.716	1.742
Middle	1.677	0.015	1.104	2.547
Fourth	2.137	0.000	1.419	3.220
Highest	2.947	0.000	1.895	4.583
**Caste/tribe**				
Scheduled tribes	1.000			
Scheduled castes	0.721	0.116	0.479	1.084
Other backward castes	0.968	0.791	0.761	1.231
General	1.090	0.547	0.824	1.443
**Religion**				
Hindu	1.000			
Muslim	0.755	0.017	0.600	0.952
Others	1.220	0.399	0.768	1.938
**Husband’s education**				
Illiterate	1.000			
Below primary	1.140	0.499	0.780	1.665
Primary but below middle	1.478	0.006	1.119	1.951
Middle but below high school	1.313	0.063	0.985	1.751
High school and above	1.563	0.003	1.162	2.102
**Mother’s education**				
Illiterate	1.000			
Below primary	1.402	0.058	0.989	1.987
Primary but below middle	1.946	0.000	1.523	2.485
Middle but below high school	1.800	0.000	1.365	2.373
High school and above	2.249	0.000	1.623	3.117
**Mother’s employment**				
Unemployed	1.000			
Professional/service/production worker	1.039	0.828	0.737	1.464
Agricultural worker/labourer	0.861	0.320	0.642	1.156
**Exposure to safe delivery messages**				
No exposure	1.000			
Only through mass media	2.052	0.000	1.414	2.977
Only through interpersonal communication	2.215	0.000	1.728	2.840
Both	2.517	0.000	1.938	3.270
**Parity**				
1	1.000			
2 +	0.509	0.000	0.414	0.626
**Full antenatal care**				
No	1.000			
Yes	2.923	0.000	2.174	3.930
**Region**				
North	1.000			
Central	1.144	0.352	0.862	1.519
North East	2.416	0.000	1.550	3.765
East	3.814	0.000	2.579	5.640
West	3.759	0.000	2.586	5.464
South	5.398	0.000	3.692	7.892

The wealth index has emerged as an important determinant of safe delivery. Adolescent mothers belonging to the fourth and highest quintiles are 2.1 (CI = 1.419–3.220) and 2.9 times (CI = 1.895–4.583), respectively, more likely to have a safe delivery compared to those belonging to the poorest wealth quintile. The likelihood of safe delivery care utilization increases with the level of the mother’s education. Compared to illiterate mothers, mothers with ten or more years of schooling are more likely to have a safe delivery (OR = 2.249, CI = 1.623–3.117). Similarly, the odds of having a safe delivery increases with the level of husband’s education.

The likelihood of safe delivery is low among Muslim mothers (OR = 0.755, CI = 0.600–0.952) compared to Hindu mothers. The odds of safe delivery among mothers of parity ‘two and above’ are lower than mothers with parity one (OR = 0.509, CI = 0.414–0.626). Adolescent mothers who have received full antenatal care during their pregnancy are almost three times more likely to have safe delivery (OR = 2.923, CI = 2.174–3.930). The odds of utilizing safe delivery care are highest in the South (OR = 5.398, CI = 3.692–7.892), followed by the West (OR = 3.759, CI = 2.586–5.464), the East (OR = 3.814, CI = 2.579–5.640) and the North-East region (OR = 2.416, CI = 1.550–3.765).

### Determinants of postnatal care utilization

Table 6 displays the odds of urban adolescent mothers receiving postnatal care in India. Wealth index, mother’s education, full antenatal care, safe delivery care and region appear as significant factors affecting postnatal care utilization. The likelihood of utilizing postnatal care is nearly two times higher (OR = 2.148, CI = 1.324–3.486) among mothers from the richest wealth quintile than among those belonging to the poorest wealth quintile. The odds of mothers with primary (OR = 1.341, CI = 1.029–1.747) and middle education (OR = 1.390, CI = 1.035–1.867) receiving postnatal care are higher than illiterate mothers. Muslim mothers are one and a half times more likely to receive postnatal care than their Hindu counterparts (OR = 1.545, CI = 1.197–1.996).

**Table 6:** Odds ratios and 95% confidence intervals (CI) for receiving postnatal care, DLHS-3 (2007–08), urban India.

Background characteristics	Odds ratio	*P*-value	95% CI	
			Lower	Upper
**Wealth quintiles**				
Lowest	1.000			
Second	1.205	0.462	0.733	1.981
Middle	1.306	0.264	0.818	2.084
Fourth	1.312	0.242	0.832	2.068
Highest	2.148	0.002	1.324	3.486
**Husband’s education**				
Illiterate	1.000			
Below primary	0.820	0.338	0.547	1.230
Primary but below middle	1.084	0.604	0.799	1.470
Middle but below high school	1.193	0.273	0.870	1.634
High school and above	1.081	0.633	0.785	1.488
**Mother’s education**				
Illiterate	1.000			
Below primary	1.280	0.199	0.878	1.866
Primary but below middle	1.341	0.030	1.029	1.747
Middle but below high school	1.390	0.029	1.035	1.867
High school and above	1.166	0.359	0.839	1.620
**Caste/tribe**				
Scheduled tribes	1.000			
Scheduled castes	0.840	0.424	0.549	1.287
Other backward castes	0.906	0.437	0.705	1.163
General	1.016	0.915	0.760	1.358
**Religion**				
Hindu	1.000			
Muslim	1.545	0.001	1.197	1.996
Others	1.119	0.634	0.704	1.780
**Mother’s employment**				
Unemployed	1.000			
Professional/service/production worker	1.029	0.875	0.723	1.463
Agricultural worker/labourer	1.115	0.500	0.813	1.530
**Parity**				
1	1.000			
2 +	0.908	0.405	0.723	1.140
**Full antenatal care**				
No	1.000			
Yes	1.900	0.000	1.452	2.488
**Safe delivery**				
No	1.000			
Yes	10.066	0.000	8.191	12.371
**Region**				
North	1.000			
Central	0.673	0.013	0.492	0.921
North East	0.553	0.009	0.353	0.865
East	0.787	0.232	0.531	1.165
West	2.432	0.000	1.609	3.676
South	2.021	0.000	1.367	2.988

The likelihood of receiving postnatal care is higher among mothers who have also received full antenatal care and safe delivery care. Those who have received full antenatal care during pregnancy are more than twice as likely to receive postnatal care than those who have not received full antenatal care (OR = 1.900, CI = 1.452–2.488). Similarly, the odds of receiving postnatal care increases by almost ten times among mothers who have opted for safe delivery compared to those who have not (OR = 10.066, CI = 8.191–12.371). Region of residence also appears as a significant factor associated with the utilization of postnatal care. Urban adolescent mothers from the North-East are less likely to receive postnatal care than their counterparts from the North (OR = 0.553, CI = 0.353–0.865). On the other hand, mothers from the South (OR = 2.0201, CI = 1.367–2.988) and the West (OR = 2.432, CI = 1.609–3.676) are almost twice more likely to receive postnatal care compared to their counterparts living in the North.

## Discussion

Maternal health care has been at the top of the agenda of the Government of India since 1996 when the integration of the Safe Motherhood and Child Health Program into the Reproductive and Child Health Program (RCH) took place. However, the issue of low utilization of maternity care among urban adolescent mothers rarely had any special place in academic and policy discussions until recently. Given the fact that the evidence on this issue in India is limited, the present study examines the factors affecting maternity care utilization among urban adolescent mothers in India. The study reveals low level of full antenatal care utilization and moderate level of safe delivery and postnatal care utilization. Several factors including education, employment status, caste, religion, wealth, and region of residence, have been found significantly associated with healthcare utilization among urban adolescent mothers.

The disparity in the use of maternal healthcare utilization across economic groups is an area of concern for many (Kumar et al., 2013; Mohanty & Pathak, 2009; Mukherjee, Singh & Chandra, 2013). Several studies have documented the fact that the household wealth has a positive effect on the use of maternal healthcare (Babalola & Fatusi, 2009; Gage, 2007; Kesterton et al., 2010; Rahman, Haque & Zahan, 2011). Our study confirms the same in the case of urban adolescent mothers in India. Mothers from richer households are more likely to use maternal care compared to mothers from the poorest households. Household wealth may facilitate the use of maternal care in many ways. Mothers from richer households are generally more educated and have more autonomy compared to mothers from the poorest households. Moreover, wealthier mothers also have enough resources to meet the expenses on healthcare whereas mothers from poor households, often less educated and unemployed, have difficulty affording their healthcare expenses. Their earnings are often so little that after spending on basic necessities of life, they are left with little or no amount of money for healthcare (Bonu et al., 2009).

Although social group as a predictor variable in case of safe delivery and postnatal care does not turn out to be statistically significant, it does emerge as a significant predictor of full antenatal care. This indicates that despite several affirmative actions by central and state governments, the caste/tribe of an individual still exerts considerable influence on healthcare utilization in urban areas (Saroha, Altarac & Sibley, 2014). In our study, SC mothers are more likely to receive full antenatal care compared to ST mothers. This is not surprising as STs are socioeconomically the most backward indigenous group of India. It is the result of their long geographical and socio-economic isolation from mainstream Indian society. In urban India, a great majority of them are migrants. They are mostly engaged in casual labour and get lowest wages among all social groups (Hall & Patrinos, 2012; Karan & Sakthivel, 2008).

Most of the studies on maternal healthcare have documented that the use of healthcare services is lower among Muslim mothers than among Hindu mothers (Pallikadavath, Foss & Stones, 2004; Salam & Siddiqui, 2006; Sanneving et al., 2013; Singh et al., 2012). In our study as well, we find that Muslim adolescent mothers are less likely to use full antenatal and safe delivery care compared to their Hindu counterparts. The Government of India set up the Sachar Committee to conduct a systematic study of the social, economic and educational status of the Muslim community. The committee concludes that Muslims ‘exhibit deficits and deprivation in practically all dimensions of development’ and ‘the deficits are particularly salient in the areas of female schooling and economic status’ (Basant, 2007; Sachar et al., 2006; Shariff, 1995). Apart from poverty and illiteracy, religious and social customs prevalent among Muslims such as ‘*burkha*/*niqab*’, physical separation of males and females, and the obligation for women to cover and hide their bodies is argued to have adverse effect on adolescent mothers’ healthcare behaviours (Singh et al., 2012). Low autonomy among Muslim mothers restricts their interaction with males outside their immediate family members (Bloom, Wypij & Gupta, 2001; Say & Raine, 2007). The presence of male doctors in hospitals thus may be an obstacle in service utilization. Muslim mothers often prefer not to go to male doctors for antenatal check-up and delivery assistance (Navaneetham & Dharmalingam, 2002).

It is surprising to see that adolescent Muslim mothers in urban areas are more likely to use the postnatal care than their Hindu counterparts. This finding is in contrast with most of the previous studies (Singh, Rai & Singh, 2012; Singh et al., 2012; Singh et al., 2014). However, a recent multilevel study in Madhya Pradesh (a state of India), conducted using the same dataset that we have used in this study, confirms the results of our study. They have not given any explanation for this anomaly (Jat, Ng & San Sebastian, 2011). The anomaly in our case can be explained by the fact that Muslim mothers in urban areas suffer more from postpartum complications than their Hindu counterparts. A simple cross-tabulation from DLHS-3 datasets suggests that the proportion of urban Muslim women, who experienced high fever (20% vs. 29%), pain in the lower abdomen (20% vs. 27%), foul vaginal smell (6.5% vs. 8.0%), excessive bleeding (8.0% vs. 10.4%), convulsions (4% vs. 5%) and severe headache (16.7% vs. 24.0%), is always higher than urban Hindu women. Hence, the prevalence of higher postpartum morbidity can be one of the reasons for greater use of healthcare services in the postnatal period by Muslim mothers. However, the relationship of religion and maternal healthcare utilization needs to be further investigated.

Similar to previous studies, the likelihood of using maternal care significantly declines among mothers with two or more children compared to those with only one child (Chakraborty, 2003; Pallikadavath, Foss & Stones, 2004; Singh, Rai & Singh, 2012). The experience, knowledge and confidence that mothers of higher parity gain from previous births is argued to be one of the main reasons behind this pattern (Navaneetham & Dharmalingam, 2002; Ochako et al., 2011). Also, the first time mother in India is often sent to her maternal home during pregnancy and childbirth. Therefore, her parents decide the kind of care she needs (Bloom, Wypij & Gupta, 2001). Parents generally provide her the best possible care during pregnancy and childbirth. Hence, it is not surprising that first time mothers are better off in terms of maternity care utilization (Navaneetham & Dharmalingam, 2002; Pallikadavath, Foss & Stones, 2004). On the other hand, women with two or more children often rely on their previous knowledge and experiences related to maternity. It is possible that they gain confidence from their experiences of previous pregnancies and births and develop a belief that modern healthcare is not necessary for them. Such mothers must be identified and provided appropriate counselling by community health workers. It must also be noted that mothers with high parity face difficulties in attending a health facility due to lack of time.

The findings of this study suggest that the use of antenatal care has a remarkable effect on the use of safe delivery care and the use of both antenatal care and safe delivery has significant effect on the use of postnatal care. Previous studies conducted in different settings have recorded similar findings (Gabrysch & Campbell, 2009; Karkee, Lee & Binns, 2013; Karkee, Lee & Khanal, 2014; Mengistu & Tafere, 2011). No statistically significant differences have been found in the use of maternal health care according to the mother’s employment.

The education of adolescent mothers is also associated with the utilization of maternal health services. These results are consistent with many studies conducted in India and other countries (Abor et al., 2011; Arokiasamy & Pradhan, 2013; Edmonds, Paul & Sibley, 2012; Haque, 2014; Kitui, Lewis & Davey, 2013; Rahman, Haque & Zahan, 2011; Saroha, Altarac & Sibley, 2014; Sharma et al., 2014; Singh, Rai & Singh, 2012; Singh et al., 2012; Singh et al., 2014; Tsegay et al., 2013; Wang, Wang & Lee, 2012; Yamashita et al., 2014). The education of the mother is argued to be an effective means of achieving greater autonomy in the family, getting employment, thereby achieving economic independence. The education also provides her opportunities to learn about pregnancy and childbirth through exposure to mass media (Acharya et al., 2010; Bloom, Wypij & Gupta, 2001). Moreover, education makes mothers confident, brings a feeling of self-worth and self-confidence, and enhances communication with their husbands and other family members on different issues including her own health (Chakraborty et al., 2002). Mothers-in-law in India generally dominate the household decision-making when it comes to issues related to female members of the household. In such circumstances, an educated woman may have an upper hand in household decision-making especially when it is about her own health needs (Navaneetham & Dharmalingam, 2002).

Similar to many previous studies, the level of the husband’s education in this study also emerges as a significant predictor in full antenatal care and delivery care utilization (Arokiasamy & Pradhan, 2013; Gabrysch & Campbell, 2009; Jat, Ng & San Sebastian, 2011; Joshi et al., 2014; Pallikadavath, Foss & Stones, 2004; Singh, Rai & Singh, 2012; Singh et al., 2012). In India, the husband’s education may contribute to maternity care utilization at least in two different ways. First, his education could help him earn enough resources to spend on his wife’s health needs. Second, his attitude towards health needs of his wife is to a certain extent is defined by his level of education. In India, a woman’s freedom of movement in public spaces is often restricted by social norms even today. In such a society, it is the attitude of a husband towards his wife’s health needs that plays an important role in maternal care utilization. Therefore, many have argued that mobilizing and involving husbands can positively contribute to improving maternal health (Chattopadhyay, 2012; Singh & Ram, 2009; Singh, Bloom & Tsui, 1998).

Urban adolescent mothers who have seen/heard/read the messages related to maternity care through mass media or interpersonal communication are more likely to utilize maternity services compared to those who have not. It is argued that the exposure to mass media results in greater awareness and dissemination of knowledge about existing programs and policies related to health care (Ankomah et al., 2014; Asp et al., 2014; Ghosh, 2006; Retherford & Mishra, 1997; Valente & Saba, 2010). Although electronic and print media has always been preferred to promote reproductive and maternal healthcare services and behaviours, interpersonal communication especially between mothers and health workers can bring significant behavioural changes in a short time. The success of grass-root level workers such as Accredited Social Health Activists (ASHA) in bringing about significant changes in health-related attitude and behaviour of the people is a good example of the effectiveness of interpersonal communication (Scott & Shanker, 2010). Since there is no information available in the dataset about the messages related to postnatal care, we have not included it in the analysis.

Regional disparities in health and healthcare utilization are often discussed (Kumar, Singh & Rai, 2013; Purohit, 2004). Our results, confirming the same, clearly illustrate regional differentials in maternity care use by urban adolescents. Urban adolescent mothers from the South and the West are more likely to use maternal health care. Higher levels of socioeconomic development and better functioning of the public health system can be some of the factors behind the better performance of the states belonging to the South and the West regions. North and Central regions include Madhya Pradesh, Rajasthan, Bihar and Uttar Pradesh (including Uttarakhand, Jharkhand and Chhattisgarh). The socioeconomic and demographic indicators of these states are poorer than the states of the West and the South (Dyson & Moore, 1983; Griffiths, Matthews & Hinde, 2002). These two regions (The North and the Central) are home to about 50% of the poor urban population of India (Planning Commission of India, 2013). The urban public health systems in these states are paralysed by huge shortages of human resource for health. For instance, according to DLHS-3 report, the proportion of Community Health Centres (CHCs) in Uttar Pradesh (also known as UP, the most populated state of India) with a gynaecologist, paediatrician, anaesthetist, health manager and blood storage facility are 50.0%, 20.8%, 16.0%, 2.7%, and 1.3%, respectively. The District Hospitals (DHs) and Urban Health Posts in the state are not in a better condition either (International Institute for Population Sciences, 2010).

## Conclusion

The present study has examined the utilization of maternal healthcare services among adolescent mothers in urban India using data from DLHS-3. The coverage of maternal healthcare, particularly full antenatal care is inadequate and far from satisfactory. Urban adolescent mothers as a group have drawn very little attention in policies and programs related to maternal health despite the fact they are among the most vulnerable groups of mothers in reproductive ages (Mangiaterra et al., 2008).

Although India has witnessed a substantial improvement in the coverage of safe delivery and postnatal care among urban adolescents, the low levels of full antenatal care utilization is still a cause for concern. One of the reasons the coverage of full antenatal care is so poor is the excessive focus of past policies and programs on expanding the coverage of safe delivery. Our study shows that the likelihood of having a safe delivery and postnatal check-up is higher among those mothers who had full antenatal care during pregnancy. This finding calls for urgent action to increase its coverage among adolescents. Ensuring full coverage of full antenatal care among adolescents can reduce adolescent maternal mortality in the country to a considerable extent. Adolescent mothers were not entitled to the benefits of *Janani Suraksha Yojana* (Mother Protection Scheme)—a conditional cash transfer program for safe motherhood until the year 2013. However, the Ministry of Health and Family Welfare has decided to extend the JSY benefits to all mothers irrespective of age and number of children. It is expected that it will help raise the levels of maternity care utilization especially that of full antenatal care among urban adolescent women. It can be considered an important step towards achieving better adolescent maternal and child health in the country (Dhar, 2013).

According to an estimate, about 50 million adolescents in India suffer from anaemia. India recently launched a nationwide Weekly Iron and Folic Acid Supplementation (WIFS) programme to combat the intergenerational cycle of anaemia in urban adolescent mothers (Kumar, 2013). Since we find that the mass media messages and interpersonal communication have a significant impact on the level of care utilization, the government should focus on promotion of maternal care services through mass media and interpersonal communication utilizing grass-root level community health workers such ASHAs (Accredited Social Health Activist) and *Anganwadi* workers.

The education of a woman and her partner exerts considerable influence on the likelihood of maternal healthcare use. We find that providing a woman only five to eight years of schooling can improve the likelihood of healthcare utilization by about 30%–95% (Fatusi & Hindin, 2010). In a low resource setting such as India, the girls often have to ‘sacrifice’ their educational opportunities for the boys of their families mainly due to higher costs of education. Although urban India has registered a significant improvement in girls’ enrolment at primary and secondary levels, high drop-out rates still pose a great threat to the efforts (Nanda et al., 2013). Educational reforms need to focus on the factors that can improve the level of education not only among the current generation of adolescent mothers but also future generations. Some of these reforms could be providing financial incentives, promoting distance education and improving educational infrastructure at school and university levels. Similarly, the education among male partners should also be encouraged as it is the males who generally have an upper hand in decision-making at household level in Indian society. Their education may lead to their greater involvement in maternal care (Chattopadhyay, 2012).

Low age at marriage is not only a barrier in adolescent woman’s education but also in her husband’s. Hence, the government should actively work towards increasing the age at marriage and providing more educational opportunities to young girls. The government should ensure proper implementation of the Prohibition of Child Marriage Act, 2006 to stop child marriages in the country (Government of India, 2007). Mothers of higher parities are less likely to use maternity care. Low contraceptive use and low age at marriage are among the main reasons behind high parity. There is a need to spread awareness about benefits of contraceptive use and higher age at marriage not only through mass media but also through active involvement of the community as these issues are intricately related with people’s cultural and religious values and beliefs.

The study reveals that socioeconomic status, caste, religion and region of residence also affect the use of maternity care among urban adolescents. These findings underscore the need to address the social determinants of health to turn urban adolescent mothers’ disadvantage in terms of maternity care utilization into an advantage (Commission on Social Determinants of Health, 2008; World Health Organization, 2011). Poor mothers in urban settings are often uneducated, unemployed and excluded from social networks. For such groups the government should have targeted interventions. In the case of Muslim adolescent mothers, where perceptions, religious beliefs and traditions affect their healthcare behaviour considerably, the health system should think of working in close collaboration with elders and religious leaders of the community (Singh et al., 2012). Apart from that, reduction in regional variation in maternal care utilization among urban adolescent mothers should also be considered a focus area for future policy and programs.

## Limitations of the study

The study has some limitations. We could not include many community and health system variables that are thought to have influence on health care utilization behaviour of mothers. DLHS-3 provides data on district hospitals’ human resources, training, equipment etc. but we could not merge that information with individual files and hence missed some important supply side variables in the analysis. Apart from these factors, local beliefs, behaviours and practices related to maternity could also not be captured in this study due to unavailability of such variables in the dataset. The study uses only three commonly used indicators of maternal health care namely full antenatal care, safe delivery and postnatal care within 42 days. In order to gain better understanding of postnatal care utilization, this study could have used two or three postnatal care indicators such as postnatal check-up within the first two days, within the first week and within the first six weeks. However, given the limited scope and space of this study, we have used only one indicator for postnatal care. Prospective studies could use more dependent variables to gain better insights into health care behaviour of urban adolescent mothers.

## Supplemental Information

10.7717/peerj.592/supp-1 Supplemental Information 1 DLHS-3 Urban
